# Interventions That Support Breastfeeding for Women With Gestational Diabetes: A Systematic Review and Meta-Analysis

**DOI:** 10.1177/10436596261417457

**Published:** 2026-02-16

**Authors:** Naema Alatawi, Rowena Ivers, Maria Mackay, Shahla Meedya

**Affiliations:** 1 University of Wollongong, New South Wales, Australia; 2 Tabuk University, Saudi Arabia; 3 Swinburne University of Technology, Melbourne, Victoria, Australia; 4 Western Sydney University, Penrith, Australia

**Keywords:** intervention, breast feeding, gestational diabetes, counseling

## Abstract

**Introduction:**

Gestational diabetes mellitus (GDM) is a common pregnancy complication associated with reduced breastfeeding rates. Breastfeeding interventions are essential in supporting women with GDM. This review evaluated the effectiveness of interventions that support breastfeeding among women with GDM.

**Methods:**

A search was conducted in PubMed, CINAHL, MEDLINE, Scopus, and CENTRAL (2000–2024). Quality was assessed using JBI checklist, and risk bias RoB2.

**Results:**

Eighteen studies were included, covering education, lifestyle support, and breastfeeding preparation. Interventions delivered by health professionals during prenatal, antenatal, and postnatal care improved exclusive breastfeeding at 6 weeks (OR 2.23, 95% CI 1.5–3.33, *p* = .005), 3–4 months (OR 2.05, CI 1.03–4.09, *p* = .04), and 6 months (OR 2.41, CI 1.48–3.93, *p* < .001), and any breastfeeding at birth and 6 months.

**Discussion:**

Educational, supportive, and culturally tailored interventions positively influenced exclusive and any breastfeeding among women with GDM. Interactive communication played a key role in breastfeeding support.

## Introduction

Gestational diabetes mellitus (GDM) is a metabolic disorder diagnosed in the second or third trimester of pregnancy in women without pre-existing diabetes and results from pregnancy-related insulin resistance driven by placental hormones (Anastasiou et al., 2020; Imoh et al., 2017; Otter et al., 2024; Quintanilla Rodriguez et al., 2025).

The prevalence of GDM is 14% across economically developed nations and is associated with adverse maternal and neonatal outcomes, including preterm birth, macrosomia, neonatal hypoglycaemia, and respiratory distress syndrome (Wang et al., 2022). In addition, mothers face a tenfold greater risk of developing type 2 diabetes, cardiovascular disease, and obesity (Ornoy et al., 2021; Preda et al., 2023). Breastfeeding supports postpartum metabolic recovery and reduces the risk of type 2 diabetes and obesity among women with GDM (Flores-Quijano et al., 2024). The recommended duration of breastfeeding is at least 6 months and has benefit when continued to 2 years of age (Meek et al., 2020; WHO, 2025).

Despite the many health benefits of breastfeeding for mothers and children, several studies indicate that mothers with GDM may face challenges in initiating and continuing breastfeeding compared to mothers without diabetes (Bever Babendure et al., 2015; Cummins, Meedya, & Wilson, 2022; Flores-Quijano et al., 2024; Nakshine & Jogdand, 2023). Furthermore, women with GDM are more likely to use formula (Doughty et al., 2018). These challenges are influenced by physiological factors, including high BMI, oedema causing nipple flattening, insulin imbalance, and prolonged labor (Bever Babendure et al., 2015).Pregnant women with GDM require special support for breastfeeding initiation and maintenance (Cummins, Meedya, & Wilson, 2022; Cummins, Wilson, & Meedya, 2022; Doughty & Taylor, 2021). (Refer to Table 1). Interventions that support breastfeeding in women with GDM vary and include nutritional support, appropriate exercise, and counseling from breastfeeding specialists (Khatib et al., 2023; Mulcahy et al., 2022; Wong et al., 2021; Wu et al., 2021).

**Table 1.** Breastfeeding Interventions.

Interventions type	Examples	Aimed	Reference
Supportive breastfeeding interventions	Educational programs.	To increase awareness of the benefits of breastfeeding, and its importance in regulating maternal and infant health	Cheng et al. (2023), Mulcahy et al. (2022)
Specialized breastfeeding	Counseling during pregnancy and after birth	To ensure a successful start	Berwick and Louis-Jacques (2023)
Practical support	Encouraging skin-to-skin contact immediately after birth	To promote early initiation of breastfeeding, and frequent feeding	Ciavarella (2023), Deys et al. (2021)

Differences in cultural and health care contexts of breastfeeding practice and support for women with GDM can influence both breastfeeding initiation and duration. The literature shows that women’s beliefs about colostrum, perceptions of milk sufficiency, and family pressures and social norms play a crucial role in breastfeeding initiation and continuation. For example, in some societies colostrum is viewed as “dirty” or “curdled milk,” leading some mothers to delay or reject it (Wanjohi et al., 2016). Qualitative studies indicate that women from diverse linguistic and cultural backgrounds face challenges in understanding GDM-related health education due to language barriers and limited cultural sensitivity among caregivers (Oxlad et al., 2023). A national survey also showed that breastfeeding support for women with GDM is often general and not tailored to their needs, and that hospitals rarely provide information on the role of breastfeeding in reducing the risk of type 2 diabetes. Regulatory and professional barriers further limit the provision of effective support for GDM (Matsunaga et al., 2021). In addition, family and social norms—such as the influence of female heads of household on breastfeeding decisions or cultural restrictions on breastfeeding in public—may limit continued breastfeeding, particularly among immigrant women or those with unequal access to care (Izumi et al., 2024). Evidence from Saudi Arabia indicates that, despite positive attitudes, initiation and exclusive breastfeeding rates remain suboptimal due to cultural norms, limited knowledge, and formula acceptance, highlighting the need for culturally responsive support (Alahmed et al., 2023). Therefore, this review aimed to assess the evidence on the effectiveness of interventions supporting breastfeeding among women with GDM, with attention to cultural and birth contexts influencing these experiences.

## Methods

A systematic review is a comprehensive analysis of available scientific evidence on a specific research question, aimed at supporting decision-making and guiding future research (Mancin et al., 2024). This review followed the Preferred Reporting Items for Systematic Reviews and Meta-Analyses (PRISMA) statement and Cochrane Collaboration guidance in reporting findings (Moher et al., 2009). The protocol was prospectively registered in the PROSPERO database (CRD42024518884), and all search and analytical procedures were conducted according to established methodological standards to ensure transparency and minimize bias.

### Search and Selection Strategy

A comprehensive literature search was conducted to identify published studies. Five databases were searched: PubMed, CINAHL, MEDLINE, Scopus, and CENTRAL. Search terms included combinations of “gestational diabetes” OR “gestational diabetes mellitus” OR “diabetes in pregnancy” OR “GDM” with “intervention,” “support,” “counselling,” “promotion,” “program,” or “educational.” Titles were imported into EndNote (version 20.6) and uploaded to Covidence, where duplicates were automatically removed. Two independent reviewers (NA and RI) screened titles and abstracts for relevance, with disagreements resolved through discussion. Potentially relevant full-text articles were then independently reviewed by the same reviewers to determine final inclusion.

### Criteria for Inclusion and Exclusion of Literature

Studies published between 2000 and 2024 in Arabic or English were included, with no restrictions on location, population, or intervention type. Eligible studies involved women with GDM and reported breastfeeding-related interventions and outcomes. Exclusion criteria comprised studies published in other languages, those involving women without GDM or with pre-existing diabetes (type 1 or type 2), and non-interventional studies or those without a breastfeeding component. Included study designs were randomized controlled trials, quasi-experimental, cohort, and cross-sectional studies.

### Quality Assessment

Quality assessment of included studies was conducted using the Joanna Briggs Institute (JBI) methodology for RCTs, quasi-experimental, cohort, and cross-sectional studies (Santos et al., 2018). Randomized controlled trials were additionally assessed using the Cochrane Risk of Bias tool (RoB 2). JBI checklist items were rated as yes, no, or not applicable, and quality scores were converted to percentages to enable comparison across study designs. Quality was categorized as low (≤33%), medium (33%–66%), or high (≥66%) (Supplementary File 1).

### Data Analysis

Data was systemically extracted from selected studies by two independent authors, with data being extracted from eligible articles by the main researcher (NA) and reviewed by the second researcher (RI). Any discrepancies identified during the review process were resolved through discussion. Extracted data was tabulated using the following variables: author, published year, country, study aim, design, and participants, breastfeeding intervention used, and breastfeeding outcomes (primary or secondary).

### Statistical Analysis

Statistical analysis was conducted using Review Manager 5.4 (Cochrane Collaboration, 2014). Fixed or random effects models were applied based on heterogeneity. Odds ratios (ORs) with 95% confidence intervals (CIs) were calculated using DerSimonian and Laird inverse variance methods. A *p* value ≤.05 indicated statistical significance. Sensitivity analyses assessed the impact of studies at high risk of bias.

## Results

### Search Findings

**Figure 1.**
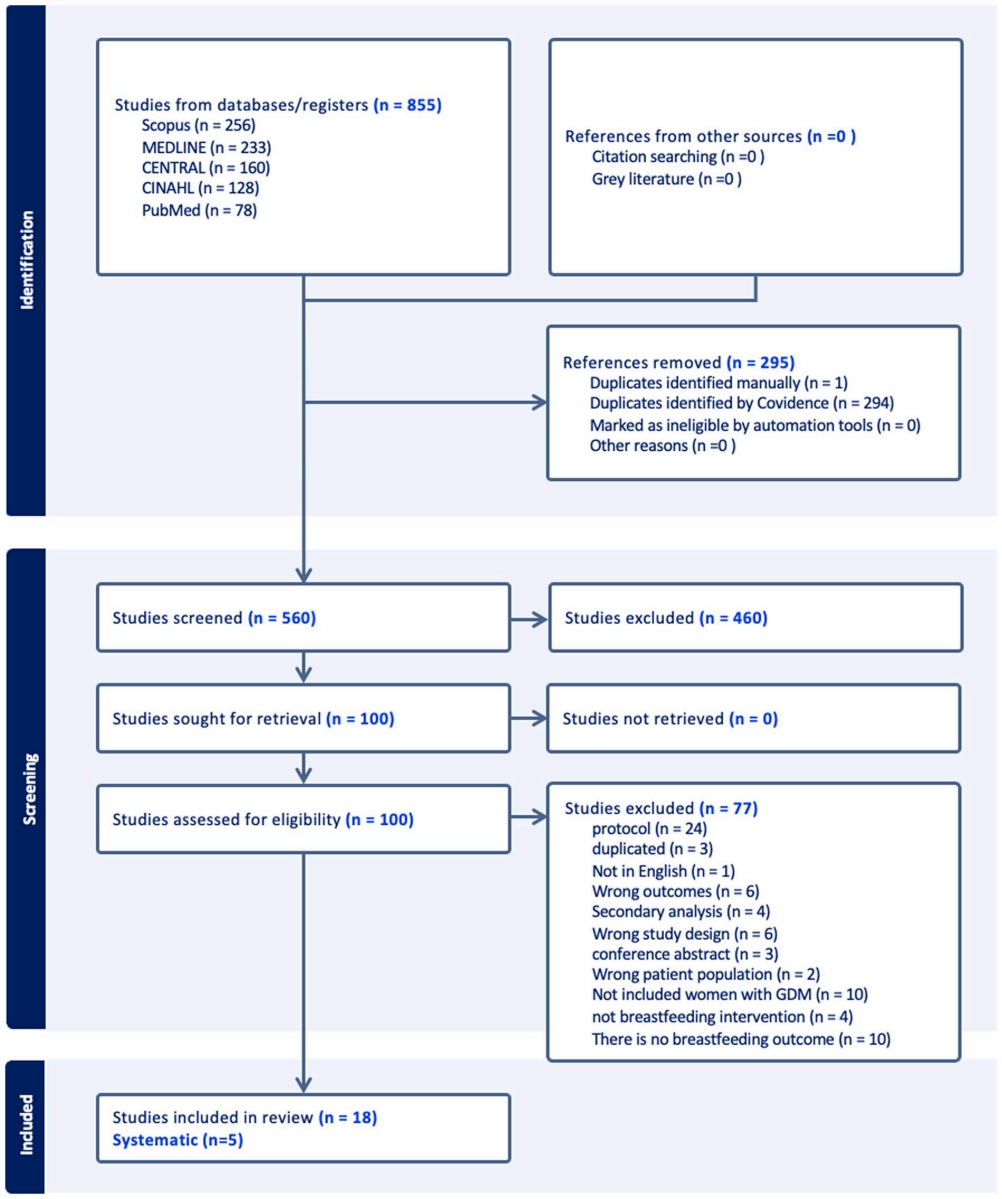
PRISMA Flowchart of the Search Strategy.

The search identified 855 records, with 295 duplicates removed. Of the remaining 560 studies, 460 were excluded for not meeting the inclusion criteria. One hundred full-text articles were assessed, of which 77 were excluded due to protocol issues, duplication, language, inappropriate outcomes or study design, secondary analysis, conference abstracts, incorrect population, or absence of breastfeeding interventions or outcomes (Figure 1). Eighteen studies were included, comprising experimental designs (RCTs, *n* = 11) and observational studies, including cohort (*n* = 3), cross-sectional (*n* = 1), and quasi-experimental designs (*n* = 3). Studies were published between 2011 and 2023 and conducted across diverse geographic regions, predominantly in high-income countries, with some from middle- and lower-resource settings. The total sample size was 6,406 participants, ranging from 28 to 2,299 per study (Table 2).

**Table 2.** The Characteristic of Included Studies (n = 18).

Author, publication year	Study aim	Country	Study design	Number of participants	Intervention used in study	Breastfeeding outcomes		Included in meta-analysis
						Primary	Secondary	
Stuebe et al. (2016)	To assess the efficacy of tailored breastfeeding (BF) support to increase BF duration among women with GDM	USA	Cluster RCT	100	Prenatal BF education, phone support, and weekly group nutrition and exercise sessions for 13 weeks postpartum	Breastfeeding (BF) duration and intensity	Introduction of formula feeding	Yes
Ferrara et al. (2011)	To evaluate the effectiveness of a pregnancy and postpartum lifestyle intervention on BF and maternal health outcomes among women with GDM	USA	RCT	197	Diet, exercise, and BF support intervention including lactation consultations and education	Postpartum weight retention	BF rates (Partial or exclusive BF)	Yes
Forster et al. (2017)	To determine the safety and efficacy of antenatal expressing of breastmilk in women with diabetes in pregnancy	Australia	Multicentre RCT	635	Antenatal expressing of breastmilk from 36 weeks vs. standard care	Neonatal Intensive Care Unit (NICU) admission rate	Exclusive BF during the initial hospital stay and at 3 months postpartum	Yes
Forster et al. (2011)	To assess the feasibility and safety of antenatal milk expressing in women with diabetes in pregnancy	Australia	Pilot RCT	43	Antenatal colostrum expressing twice daily from 36 weeks of gestation, with instructions on safe storage	Infant feeding outcomes	Maternal and infant well-being	No
You et al. (2020)	To evaluate the effectiveness of BF education based on self-efficacy theory on BF outcomes in women with GDM	China	RCT	226	Perinatal individualized BF education based on self-efficacy theory, including 4 phases of education and support led by IBCLC	BF self-efficacy and exclusive BF	Any BF rates	Yes
Gilbert et al. (2023)	To assess the impact of a pre- and postpartum multidimensional lifestyle and psychosocial intervention on offspring outcomes in women with GDM	Australia	Single-blind RCT,	211	MySweetheart trial Intervention included lifestyle and psychosocial support focusing on diet, physical activity, mental health, and BF support	Maternal outcomes	BF, offspring’s birth, anthropometric, and psycho behavioral outcomes	Yes
Truva et al. (2021)	To investigate the effect of a structured individualized lactation educational intervention on increasing BF rates among women with endocrine disorders and low-risk women	Greece	RCT	200	Structured educational intervention by midwife, including prenatal and postnatal BF support	Duration of BF	Exclusive BF rates	Yes
Ural and Kizilkaya Beji (2021)	To investigate the effects of the Health-Promoting Lifestyle Education Program on maternal lifestyle, quality of life, depression symptoms, and neonatal health among women with GDM	Turkey	RCT	88	Health-Promoting Lifestyle Education Program consisting of three 45-minute sessions on self-management, nutrition, physical activity, stress management, and BF support	Maternal quality of life, lifestyle behaviors	Neonatal health (e.g., macrosomia, BF rates)	Yes
Parsons et al. (2022)	To assess the feasibility of an ante- and postnatal lifestyle intervention for women with GDM to reduce type 2 diabetes risk	UK	Partially randomized feasibility controlled trial	50	Four motivational interview-based sessions (two antenatally, two postnatally), WhatsApp support, FitBit, and electronic resources	Weight loss	BF, blood glucose, physical activity	Yes
Tawfik (2017)	To investigate the impact of a health belief model (HBM)-based educational intervention on knowledge, beliefs, and self-reported practices among women with GDM	Egypt	Cluster RCT	201	HBM-based health education intervention focusing on knowledge, risk perception, and self-management vs. usual care	Knowledge and beliefs scores	Exclusive BF rates, postpartum weight retention	Yes
Johnsen et al. (2021)	To determine the feasibility, practicality, and acceptability of antenatal breastmilk expression (ABE) among Scandinavian women with diabetes in pregnancy	Norway	Observational feasibility study	28	Participants received instructions on ABE starting from week 37+0, continued until hospital admission for birth, and collected colostrum	BF rates at discharge	Infant hypoglycemia, NICU admission	No
Dalsgaard et al. (2019)	To investigate the effect of early, frequent BF and skin-to-skin contact on neonatal blood glucose levels among infants born to mothers with diet-treated GDM	Denmark	Quasi-experimental study with a historical control group	533	Early, frequent BF and two hours of immediate uninterrupted skin-to-skin contact following birth	Neonatal hypoglycemia incidence	BF frequency within first six hours postpartum	Yes
Cai et al. (2020)	To determine the effect of comprehensive nursing on blood glucose level, unhealthy emotions, and pregnancy outcomes in women with GDM	China	Quasi-experimental study	159	Comprehensive nursing including psychological support, dietary guidance, exercise intervention, and life management	Blood glucose levels, pregnancy outcomes	Postpartum depression, BF self-efficacy	No
Mustafa et al. (2022)	To assess the impact of adherence to dietary guidelines on maternal and infant health in women with GDM	New Zealand	Cohort study	313	Assessment of adherence to dietary guidelines; participants scored on adherence levels (high, moderate, low	Adherence impact on maternal and neonatal health	BF rates at hospital discharge	No
Schellinger et al. (2017)	To determine the impact of Centering Pregnancy© group prenatal care on pregnancy outcomes and postpartum follow-up in Hispanic women with GDM	USA	Retrospective Cohort study	460	Centering Pregnancy© group prenatal care model with facilitated group sessions, education, and peer support vs. traditional care	Postpartum glucose tolerance testing	BF initiation at discharge and BF rate at postpartum	Yes
Griffin et al. (2022)	To evaluate the effect of lactation consultation by an IBCLC on breastfeeding rates among mothers with GDM	USA	Retrospective Cohort study	517	IBCLC consultation during postpartum hospitalization vs. no consultation	Any BF at discharge and 3 months postpartum	Exclusive BF rates	Yes
Loewenberg Weisband et al. (2017)	To assess the associations between GDM, exclusive BF intention, hospital supplementation, and breastfeeding duration	USA	Cross sectional study	2299	Analysis of BF intention, hospital supplementation, and duration by GDM history; mediation analysis conducted	BF duration	Intention to exclusively BF, hospital supplementation	No
Cohen et al. (2023)	To assess whether an educational video clip presented by a multidisciplinary team could increase BF rates among women with GDM	Israel	Prospective double-blind RCT	146	Educational video clip presented by a team including an obstetrician, pediatrician, dietitian, and certified breastfeeding consultant	BF rates postpartum	Intention to BF	No

### Intervention Characteristics

All 18 studies reported on breastfeeding interventions during perinatal care (prenatal, antenatal, and postnatal) among women with GDM.

All studies included involved providing informational support (*n* = 18) (Cai et al., 2020; Cohen et al., 2023; Dalsgaard et al., 2019; Ferrara et al., 2011; Forster et al., 2011, 2017; Gilbert et al., 2023; Griffin et al., 2022; Johnsen et al., 2021; Loewenberg Weisband et al., 2017; Mustafa et al., 2022; Parsons et al., 2022; Schellinger et al., 2017; Stuebe et al., 2016; Tawfik, 2017; Truva et al., 2021; Ural & Kizilkaya Beji, 2021; You et al., 2020), with some providing motivation for self-management (*n* = 5) (Forster et al., 2011, 2017; Tawfik, 2017; Ural & Kizilkaya Beji, 2021; You et al., 2020), relaxation techniques (*n* = 3) (Forster et al., 2017; Ural & Kizilkaya Beji, 2021; You et al., 2020), and emotional support (*n* = 4) (Forster et al., 2017; Parsons et al., 2022; Tawfik, 2017; You et al., 2020).

Intervention providers included lactation consultants (*n* = 6) (Dalsgaard et al., 2019; Ferrara et al., 2011; Forster et al., 2017; Griffin et al., 2022; Stuebe et al., 2016; You et al., 2020), obstetrician (*n* = 2) (Cohen et al., 2023; Dalsgaard et al., 2019), pediatrician (*n* = 2) (Cohen et al., 2023; Dalsgaard et al., 2019), dietitian (*n* = 5) (Cohen et al., 2023; Ferrara et al., 2011; Gilbert et al., 2023; Mustafa et al., 2022; Ural & Kizilkaya Beji, 2021), nurses (*n* = 6) (Cai et al., 2020; Cohen et al., 2023; Gilbert et al., 2023; Loewenberg Weisband et al., 2017; Parsons et al., 2022; Ural & Kizilkaya Beji, 2021), midwife (*n* = 3) (Forster et al., 2017; Gilbert et al., 2023; Truva et al., 2021), health educator (*n* = 1) (Schellinger et al., 2017), and physicians (*n* = 6) (Forster et al., 2011; Gilbert et al., 2023; Johnsen et al., 2021; Mustafa et al., 2022; Schellinger et al., 2017; Stuebe et al., 2016; Ural & Kizilkaya Beji, 2021).

Interventions for breastfeeding were performed either within hospital or other settings. A variety of interventions methods were used: telephone communication (*n* = 9) (Cohen et al., 2023; Dalsgaard et al., 2019; Ferrara et al., 2011; Forster et al., 2011, 2017; Griffin et al., 2022; Stuebe et al., 2016; Truva et al., 2021; Ural & Kizilkaya Beji, 2021), text messages (*n* = 3) (Gilbert et al., 2023; Parsons et al., 2022; Stuebe et al., 2016) WhatsApp (*n* = 1) (Parsons et al., 2022) and WeChat (*n* = 1) (You et al., 2020), or consultations (*n* = 3) (Dalsgaard et al., 2019; Johnsen et al., 2021; You et al., 2020), routine clinic visit (*n* = 4) (Gilbert et al., 2023; Mustafa et al., 2022; Tawfik, 2017; You et al., 2020), activities (*n* = 3) (Gilbert et al., 2023; Schellinger et al., 2017; Ural & Kizilkaya Beji, 2021), awareness sessions (*n* = 1) (Schellinger et al., 2017), or health education using videos (*n* = 5) (Cohen et al., 2023; Dalsgaard et al., 2019; Ferrara et al., 2011; Mustafa et al., 2022; Schellinger et al., 2017), handbooks (*n* = 8) (Dalsgaard et al., 2019; Ferrara et al., 2011; Forster et al., 2011, 2017; Griffin et al., 2022; Mustafa et al., 2022; Schellinger et al., 2017; You et al., 2020), and website (*n* = 1) (Parsons et al., 2022).

Intensity of the intervention was reported for 7 out of 18 studies and included up to four sessions for 2 hours (Schellinger et al., 2017), three sessions of 45 minutes, for 3 consecutive days (Ural & Kizilkaya Beji, 2021), two sessions for 1 hour each (Parsons et al., 2022) or one session (Ferrara et al., 2011; Forster et al., 2011, 2017; Johnsen et al., 2021).

Most of included studies used individual interventions (Cai et al., 2020; Dalsgaard et al., 2019; Ferrara et al., 2011; Forster et al., 2011, 2017; Griffin et al., 2022; Johnsen et al., 2021; Loewenberg Weisband et al., 2017; Mustafa et al., 2022; Parsons et al., 2022; Tawfik, 2017; Truva et al., 2021; Ural & Kizilkaya Beji, 2021; You et al., 2020). Group interventions were used in three studies (Cohen et al., 2023; Schellinger et al., 2017; Stuebe et al., 2016). A combination of individual and group interventions was used in one study (Gilbert et al., 2023).

The time of intervention varied among included studies: prenatal (Cohen et al., 2023; Forster et al., 2011, 2017), during pregnancy (Johnsen et al., 2021; Mustafa et al., 2022; Schellinger et al., 2017; Truva et al., 2021; Ural & Kizilkaya Beji, 2021), immediately after birth (Dalsgaard et al., 2019), during pregnancy and continuing after birth (Cai et al., 2020; Ferrara et al., 2011; Gilbert et al., 2023; Loewenberg Weisband et al., 2017; Stuebe et al., 2016; Tawfik, 2017; You et al., 2020), and postpartum only (Griffin et al., 2022).

The studies included diverse populations, including a range of cultural and ethnic backgrounds and a range of health care settings. Few studies focussed on incorporating specific cultural factors into design or delivery of interventions. Some studies targeted particular sub-populations, such as Hispanic women (Schellinger et al., 2017), while others included women receiving lactation consultations in culturally diverse hospitals; however,Griffin et al. (2022) did not report on outcomes in different populations. Moreover, status as Baby Friendly Hospitals was not reported; the Baby Friendly Hospital Initiative supports culturally safe care delivery (Bacciaglia & Neufeld, 2022). Therefore, this characteristic could not be extracted.

However, there was consistency across the effect of interventions from lower-middle to high-income countries, and in English and non-English speaking countries, including Greece (Truva et al., 2021), Turkey (Ural & Kizilkaya Beji, 2021), Egypt (Tawfik, 2017), and China (You et al., 2020), demonstrating the universality of women’s experience in breastfeeding.

### Types of Breastfeeding Interventions

#### Educational Interventions

Two studies reported on the use of educational interventions to support breastfeeding (Stuebe et al., 2016; You et al., 2020). These interventions included: nutrition, exercise and coping skills training (NEST), and International Board Certified Lactation Consultant (IBCLC) programs. NEST provided tailored postpartum breastfeeding education in pre- and postnatal period, while the IBCLC intervention promoted self-efficacy (Stuebe et al., 2016; You et al., 2020). Two studies focused on breastfeeding education interventions, including structured midwifery breastfeeding education, and video clips developed by multidisciplinary team (Cohen et al., 2023; Truva et al., 2021). A prenatal care intervention was reported with Schellinger et al. (2017) using the centring pregnancy© group prenatal care model delivered via facilitated group sessions.

#### Lifestyle Interventions

Some studies assessed lifestyle interventions within perinatal care. The MySweetheart model provided breastfeeding education alongside psychological and healthy lifestyle support (Gilbert et al., 2023), while the Diet, Exercise and Breastfeeding Intervention (DEBI) delivered a postpartum lifestyle program (Ferrara et al., 2011). A health-promoting lifestyle education program addressed self-management, nutrition, physical activity, stress management, and breastfeeding support (Ural & Kizilkaya Beji, 2021). Nursing interventions included psychological support, dietary guidance, exercise, and life management (Cai et al., 2020; Mustafa et al., 2022). Immediate uninterrupted skin-to-skin contact after birth also improved breastfeeding outcomes (Dalsgaard et al., 2019).

Parsons et al. (2022) used the GODDESS program with four motivational interview-based sessions to support lifestyle change. Similarly, Tawfik (2017) applied a health belief model–based educational intervention on knowledge, beliefs, and practices among women with GDM. In these lifestyle-focused studies, breastfeeding was assessed as a secondary outcome.

### Interventions for Preparation for Lactation

Interventions included postpartum consultation with an IBCLC during hospital admission (Griffin et al., 2022). Two studies involved interventions involving milk expression during pregnancy. Johnsen et al. (2021) reported the feasibility and acceptability of antenatal breastmilk expression (ABE) to collect colostrum, while Forster et al. (2011) advised antenatal colostrum expression with guidance on safe storage. The efficacy and safety of ABE from 36 weeks’ gestation were later assessed (Forster et al., 2017). Another intervention examined the impact of hospital supplementation with water, infant formula, or sugar water on breastfeeding intention and duration (Loewenberg Weisband et al., 2017).

### Intervention Outcomes

Outcomes included breastfeeding at discharge and three months after birth (*n* = 4) (Schellinger et al., 2017; Griffin et al., 2022; Johnsen et al., 2021; Mustafa et al., 2022). Other outcomes included breastfeeding duration (*n* = 4) (Cohen et al., 2023; Stuebe et al., 2016; Truva et al., 2021; Loewenberg Weisband et al., 2017), rates (*n* = 1) (Ferrara et al., 2011), any breastfeeding rates (*n* = 2) (Cohen et al., 2023; You et al., 2020), exclusive breastfeeding rates (*n* = 6) (Forster et al., 2017; Griffin et al., 2022; Tawfik, 2017; Truva et al., 2021; Loewenberg Weisband et al., 2017; You et al., 2020), adherence to exclusive breastfeeding (*n* = 1) (You et al., 2020), intensity (*n* = 1) (Stuebe et al., 2016), and self-efficacy (*n* = 2) (Cai et al., 2020; You et al., 2020). Some studies measured infant feeding outcomes, including infant feeding (*n* = 1) (Forster et al., 2011), postnatal weight retention (*n* = 1) (Ferrara et al., 2011), neonatal intensive care unit admission rates (*n* = 2) (Forster et al., 2017; Johnsen et al., 2021), and incidence of hypoglycemia (*n* = 2) (Dalsgaard et al., 2019; Johnsen et al., 2021).

Maternal outcomes were reported in seven studies, including lifestyle behaviors (Ural & Kizilkaya Beji, 2021), weight loss (Parsons et al., 2022), blood glucose levels, and postpartum depression, and breastfeeding self-efficacy (Cai et al., 2020; Schellinger et al., 2017), knowledge and beliefs about health and nutrition (Tawfik, 2017), anthropometric and psychological outcomes (Gilbert et al., 2023) and maternal and infant well-being (Forster et al., 2011; Ural & Kizilkaya Beji, 2021).

### Study Quality

**Figure 2.**
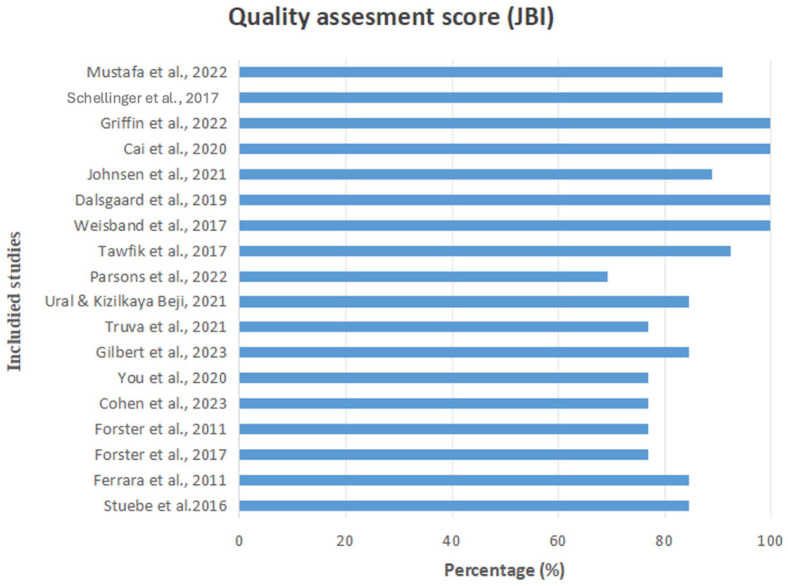
Quality Assessment Score of Included Studies Using JBI Checklist.

Overall, the quality assessment scores of the included studies were high according to the JBI checklist. Scores ranged from 69.2% to 92.3% for RCTs, 88.9% to 100% for quasi-experimental studies, 90.9% to 100% for cohort studies, and 100% for the cross-sectional study (Figure 2). Quality evaluation showed that researchers and participants were generally aware of the interventions received, except in Tawfik (2017), which was blinded for participants only. This awareness may introduce potential bias in outcomes based on subjective measures, such as self-reported breastfeeding challenges. Non-RCT studies were assessed using tools with established reliability and validity, with appropriate follow-up, intervention administration, outcome measurement, and consideration of statistical validity.

**Figure 3.**
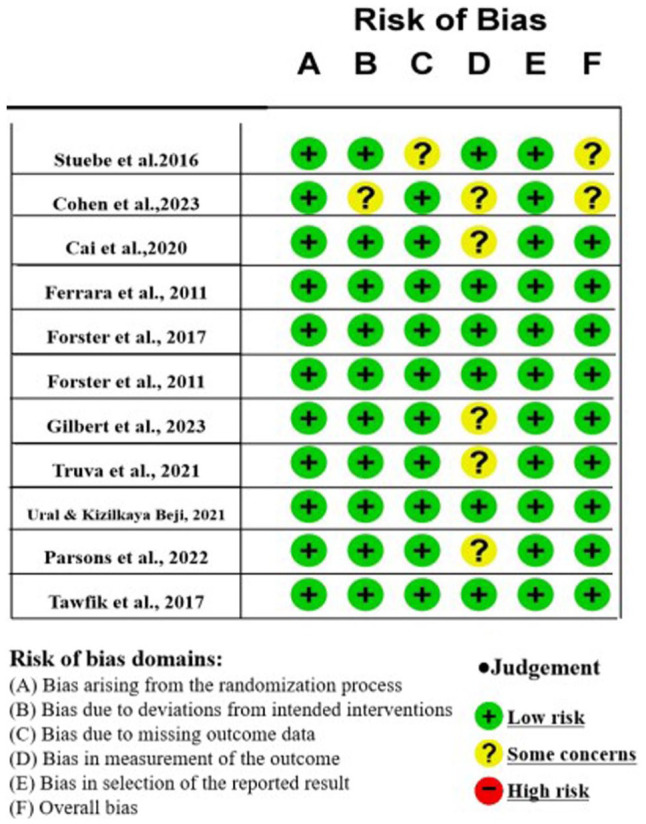
Quality Assessment of RCTs (n = 11)*Using RoB2.*

Nine out of 11 of RCTs studies had low risk of bias, except Stuebe et al. (2016) in which there was some bias, which also appeared to have missing outcome data. In the other studies, measurement of the outcome was not clear (Gilbert et al., 2023; Parsons et al., 2022; Truva et al., 2021). Otherwise, the overall quality of the nine RCTs studies remained relatively high, with most of them demonstrating adequate randomization process, no deviations from intended interventions with no missing outcome and well-chosen outcome (Figure 3).

### Findings

The effect of intervention meta-analysis included 11 studies, including RCTs (*n* = 8), cohort (*n* = 2), and quasi-experimental study (*n* = 1) for any breastfeeding or exclusive breastfeeding. The time points of breastfeeding varied among included studies.

**Figure 4.**
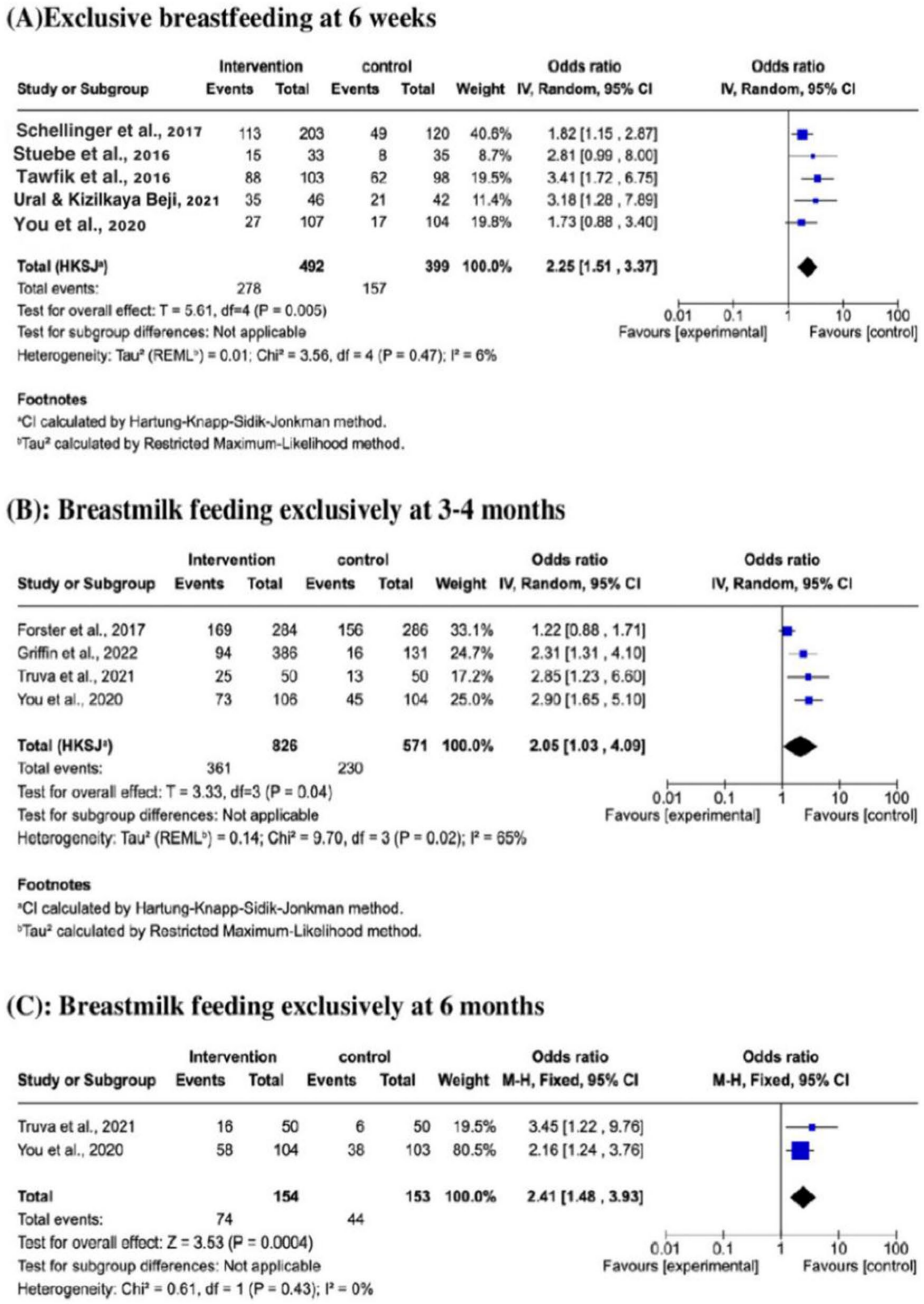
Forest Plots of Exclusive Breastfeeding Outcomes Reported at (A) 3–6 Weeks, (B) 4–6 Months, and (C) 6 Months.

Exclusive breastfeeding outcome analysis showed that a breastfeeding support intervention, compared with the control group, increased the likelihood of mothers continuing exclusive breastfeeding for 6 weeks, 3–4 months, and 6 months by 2.23, 2.05, and 2.41 times with odds ratio (OR) = 2.23, 95% confidence interval (CI) = 1.5–3.33), *p* = .005, OR = 2.05, CI: 1.03–4.09, *p* = .04, OR = 2.41, CI: 1.48–3.93, *p* = .0004, respectively, as shown in Figure 4.

Heterogeneity between studies reported at 6 weeks and 6 months was low (I^2^ = 6%, and 0%) enhancing the reliability of the positive effects of the intervention (Figure 4A and C). However, studies reported for 3–4 months had moderate to high heterogeneity (I^2^ = 65%).

**Figure 5.**
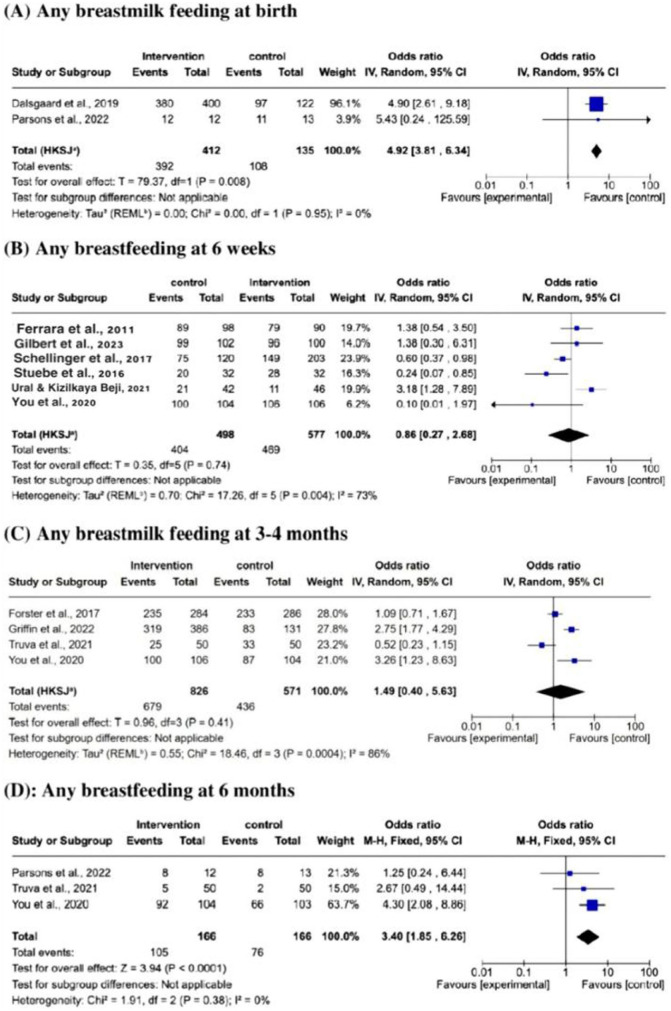
Forest Plots of Any Breastfeeding Outcomes at (A) Birth, (B) 6 Weeks, (C) 3–4 Months, and (D) 6 Months

For any breastfeeding, the breastfeeding support intervention, compared with the control group, increased the likelihood of mothers at birth and 6 months by 4.92 and 3.40 times, respectively (OR = 4.92, CI = 3.81–6.34, *p* = .008; OR = 3.40, CI = 1.85–6.26, *p* <.0001) with low heterogeneity at birth and 6 months (I^2^ = 0%). No differences were seen for the 6 weeks, and 3-4 months’ time points, with high heterogeneity (I^2^=73%, and 86%) (Figure 5).

### Sensitivity Analysis

**Figure 6.**
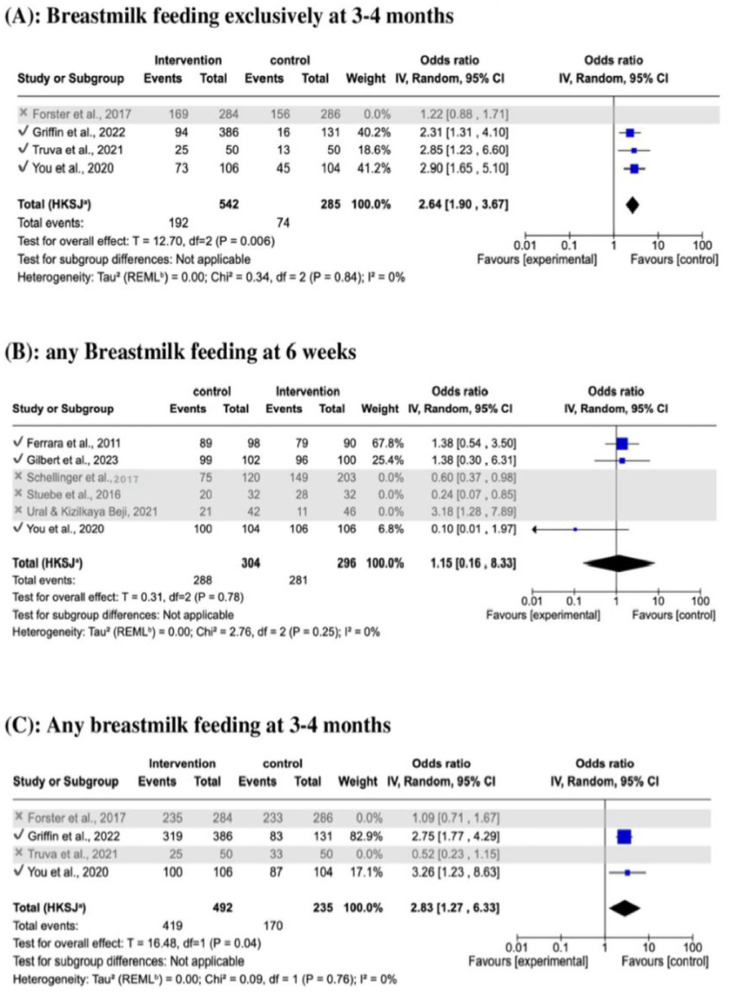
Sensitivity Analysis of Breastmilk Feeding Exclusively at (A) 3–4 Months and Any Breastfeeding Outcomes, (B) 6 Weeks, and (C) 3–4 Months

Sensitivity analysis (Jpt, 2008) was conducted to exclude high risk of bias studies, including exclusive breastfeeding at 3–4 months and any breastfeeding at 2 points of time, 3–4 months and 6 months, to excluded high risk of bias studies. The OR of exclusive breastfeeding increased by 59% at 3–4 months for the educational intervention group (OR 2.64, CI = 1.90–3.67, *p* = .006) compared with the control group. For any breast feeding at 6 weeks and 3–4 months, respectively, the OR was over 49 and 29 times greater than that of the control group (Figure 6).

## Discussion

This is the first systematic review that has been carried out specifically on women with GDM from different cultures that aims to synthesize the effectiveness of breastfeeding support interventions in women with GDM. The research question was: “What is the effectiveness of interventions to support breastfeeding among women with gestational diabetes mellitus?”. The pooled results indicate educational programs were the most effective interventions across different studies. Providing substantial evidence to support the implementation of this intervention in clinical practice to improve breastfeeding in pregnant women with GDM.

The meta-analysis results showed that supportive breastfeeding interventions in women with GDM could improve breastfeeding rates. There were several key findings: Improved exclusive breastfeeding rates during the first months postpartum. This effectiveness appears to stem from empowering mothers through structured education and healthy lifestyle promotion delivered directly and interactively which fosters self-confidence and self-efficacy and encourages commitment to exclusive breastfeeding (Schellinger et al., 2017; Tawfik, 2017; Ural & Kizilkaya Beji, 2021).

Evidence also suggests that educational programs designed to meet the needs of all mothers, regardless of their GDM status, can be broadly effective. This may be because these programs give mothers sufficient time to prepare, develop their knowledge, and build awareness and skills. Ongoing follow-up also enhances mothers’ self-confidence, which supports continued breastfeeding. Combining antenatal education with practical postnatal interventions appears to promote sustained exclusive breastfeeding. This is a need that has been requested by women in a qualitative study among 13 women who provided their feedback on parenting educational classes (Hassanzadeh et al., 2021).

The results demonstrate that educational counseling before and during pregnancy was effective in supporting breastfeeding continuity in women with GDM for 3–4 months (Griffin et al., 2022; Truva et al., 2021; You et al., 2020). These interventions focused on early education and awareness-raising through support from midwives and counselors, with direct midwifery support enhancing mothers’ confidence in their breastfeeding abilities. Training mothers in pumping techniques was particularly effective in situations of mother–infant separation or return to work (Li et al., 2024). Furthermore, women with GDM were more likely to continue exclusive breastfeeding for 6 months with IBCLC support and structured midwifery breastfeeding education programs (Truva et al., 2021; You et al., 2020). One IBCLC-delivered intervention based on self-efficacy theory supported mothers across multiple breastfeeding stages through education, counseling, and follow-up, enabling continued breastfeeding for 6 months by strengthening maternal confidence and skills (You et al., 2020).

Uninterrupted skin-to-skin contact following birth stimulates the infant’s natural suckling response and increases early breastfeeding, including colostrum intake (Dalsgaard et al., 2019). It also enhances maternal satisfaction and bonding, particularly after surgical birth (Deys et al., 2024). Many interventions combined multiple approaches.

The greatest improvements in breastfeeding rates were observed in interventions delivered by lactation consultants, nurses, or physicians (Cai et al., 2020; Cohen et al., 2023; Dalsgaard et al., 2019; Ferrara et al., 2011; Forster et al., 2017; Gilbert et al., 2023; Griffin et al., 2022; Johnsen et al., 2021; Loewenberg Weisband et al., 2017; Mustafa et al., 2022; Parsons et al., 2022; Stuebe et al., 2016; Truva et al., 2021; Ural & Kizilkaya Beji, 2021; You et al., 2020). Collaboration between these professionals provides comprehensive support and increases breastfeeding. Our findings align with WHO and UNICEF guidelines and other reviews, such as Rifat et al. (2024), which emphasize the importance of breastfeeding support in maternal and newborn care facilities. These interventions involved high levels of communication, either face-to-face or via telephone, highlighting the importance of regular support. Similar benefits have also been reported among women without GDM (Whittaker et al., 2025).

The effectiveness of breastfeeding support was influenced by cultural and health system contexts. For instance, a quantitative study in Hispanic women in the United States highlighted the role of family and peer involvement through group prenatal care (Schellinger et al., 2017). In China, interventions based on self-efficacy theory were effective, though postpartum confinement traditions sometimes delayed initiation (You et al., 2020). Across studies in many nations and cultures, interventions relied on structured face-to-face education and multidisciplinary support (Cohen et al., 2023; Tawfik, 2017; Ural & Kizilkaya Beji, 2021), reflecting the importance of direct personal counseling for all women. By contrast, European digital interventions such as Pregnant+ and MySweetheart highlighted challenges with digital literacy, and engagement (Borgen et al., 2019; Gilbert et al., 2023). This suggests that in lower-resource settings, where internet coverage, and smartphone ownership may be limited, reliance on digital interventions could create additional barriers to equitable uptake. These cultural considerations highlight the importance of tailoring interventions to local norms, family structures and supporting the delivery of face-to-face support to women while considering system-level capacity. There is also a need to consider beliefs about colostrum, perceptions of milk adequacy, the influence of family and breastfeeding norms so as to be effective in promoting sustained exclusive breastfeeding. Nurses and health care providers should incorporate cultural awareness, involve family members when appropriate, and adapt educational strategies to align with the mother’s cultural context, thereby fostering confidence and adherence to recommended breastfeeding practices.

Most studies that improved exclusive breastfeeding included two to four antenatal sessions (Schellinger et al., 2017; Ural & Kizilkaya Beji, 2021), consistent with McFadden et al. (2019), while Reichental et al. (2022) suggested 3–11 sessions. This review found that educational supportive programs were most effective in improving exclusive and any breastfeeding, consistent with previous meta-analyses (Guise et al., 2003; Jung et al., 2021). Individual interventions were more effective than group approaches at birth, 6 weeks, 3–4 months, and 6 months (Jung et al., 2021; Reichental et al., 2022).

Interventions positively influenced breastfeeding initiation and duration. These primary outcomes were complemented by secondary outcomes that included improved breastfeeding technique, fewer breastfeeding problems, maternal and infant health effects, and mothers’ satisfaction with support. Some studies also examined whether interventions to prevent type 2 diabetes supported breastfeeding (Parsons et al., 2022; Tawfik, 2017).

This review included studies with varied designs. The inclusion of RCTs enabled meta-analysis and strengthened the validity of the findings. Due to the limited studies on this topic, other designs were included to provide a broader view of practice. The findings highlight the need for future research using multi-stage individual interventions across the perinatal period, with standardized criteria for analysis, including session number, timing, breastfeeding timepoints, blinding, and Baby Friendly Hospital designation.

### Study Limitations

Despite the low risk of bias in most studies, the quality of some studies was of concern. Lack of double blinding and selection bias were major limitations, for example, a control group with individual interventions but an intervention group with group interventions (Schellinger et al., 2017). In addition, participants who declined randomization in one study were allowed to choose their assigned group, with personal preference influencing group allocation (Parsons et al., 2022). The limited number of studies for certain outcomes restricted subgroup analyses and comparability. Furthermore, the review was limited to studies published in Arabic and English. Variation in intervention definitions and intensity reduced Generalizability across health care systems and cultures. Finally, potential confounding factors cannot be ruled out despite efforts to minimize them through rigorous methodological standards.

## Conclusion

This study, a systematic review and meta-analysis, investigated the effectiveness of interventions to support breastfeeding among women with GDM. The findings suggest that educational supportive breastfeeding interventions positively impact both exclusive, and any breastfeeding rates for women with GDM, encouraging them to continue breastfeeding for at least 6 months.

These findings underscore the importance of targeted, and supportive and culturally tailored breastfeeding interventions, especially educational programs with interactive communication, in improving breastfeeding outcomes for women with GDM.

## Supplemental Material

sj-docx-1-tcn-10.1177_10436596261417457 – Supplemental material for Interventions That Support Breastfeeding for Women With Gestational Diabetes: A Systematic Review and Meta-Analysis Supplemental material, sj-docx-1-tcn-10.1177_10436596261417457 for Interventions That Support Breastfeeding for Women With Gestational Diabetes: A Systematic Review and Meta-Analysis by Naema Alatawi, Rowena Ivers, Maria Mackay and Shahla Meedya in Journal of Transcultural Nursing
