# Supplementary material for: Interventions That Support Breastfeeding for Women With Gestational Diabetes: A Systematic Review and Meta-Analysis
Source: J Transcult Nurs. 2026 Feb 16;37(4):625–40. doi: 10.1177/10436596261417457 (PMC13276113; doi:10.1177/10436596261417457)
Supplement: sj-docx-1-tcn-10.1177_10436596261417457 – Supplemental material for Interventions That Support Breastfeeding for Women With Gestational Diabetes: A Systematic Review and Meta-Analysis [file sj-docx-1-tcn-10.1177_10436596261417457.docx]

**Appendix (1) : JBI checklist**

| **JBI checklist for Randomized Ccontrolled Trials (RCTs)** | | | | | | | | | | | |
| --- | --- | --- | --- | --- | --- | --- | --- | --- | --- | --- | --- |
|  | **Tawfik et al., 2017** | **Parsons et al., 2022** | **Ural & Kizilkaya Beji, 2021** | **Truva et al., 2021** | **Ferrara et al., 2011** | **Gilbert et al., 2023** | **Forster et al., 2011** | **Forster.,et al 2017** | **Cohen et al., 2023** | **You et al., 2020** | **Stuebe et al.2016** |
| 1. Was true randomization used for assignment of participants to treatment groups? | Yes | Yes | Yes | Yes | Yes | Yes | Yes | Yes | Yes | Yes | Yes |
| 2. Was allocation to treatment groups concealed? | Yes | Yes | Yes | Yes | Yes | Yes | Yes | Yes | Yes | Yes | Yes |
| 3. Were treatment groups similar at the baseline? | Yes | Yes | Yes | Yes | Yes | Yes | Yes | Yes | Yes | Yes | Yes |
| 4. Were participants blind to treatment assignment? | Yes | No | No | No | No | No | No | No | No | No | No |
| 5. Were those delivering treatment blind to treatment assignment? | No | No | No | No | No | No | No | No | No | No | Np |
| 6. Were outcomes assessors blind to treatment assignment? | Yes | Unclear | Yes | Unclear | Yes | Yes | Yes | Yes | No | Yes | Yes |
| 7. Were treatment groups treated identically other than the intervention of interest? | Yes | Yes | Yes | Yes | Yes | Yes | Yes | Yes | Yes | Yes | Yes |
| 8. Was follow up complete and if NOt, were differences between groups in terms of their follow up adequately described and analyzed? | Yes | No | Yes | Yes | Yes | Yes | Unclear | Unclear | Yes | No | Yes |
| 9. Were participants analyzed in the groups to which they were randomized? | Yes | Yes | Yes | Yes | Yes | Yes | Yes | Yes | Yes | Yes | Yes |
| 10. Were outcomes measured in the same way for treatment groups? | Yes | Yes | Yes | Yes | Yes | Yes | Yes | Yes | Yes | Yes | Yes |
| 11. Were outcomes measured in a reliable way? | Yes | Yes | Yes | Yes | Yes | Yes | Yes | Yes | Yes | Yes | Yes |
| 12. Was appropriate statistical analysis used? | Yes | Yes | Yes | Yes | Yes | Yes | Yes | Yes | Yes | Yes | Yes |
| 13. Was the trial design appropriate, and any deviations from the standard RCT design (individual randomization, parallel groups) accounted for in the conduct and analysis of the trial? | Yes | Yes | Yes | Yes | Yes | Yes | Yes | Yes | Yes | Yes | Yes |

| **JBI checklist for cross sectional study** | | **JBI checklist for quasi-experimental studies** | | | | **JBI checklist for cohort studies** | | | |
| --- | --- | --- | --- | --- | --- | --- | --- | --- | --- |
|  | Weisband et al., 2017 |  | Dalsgaard et al., 2019 | Johnsen et al., 2021 | Cai et al., 2020 |  | Griffin et al., 2022 | Schellinger et al., 2017 | Mustafa et al., 2022 |
| 1.Were the criteria for inclusion in the sample clearly defined? | Yes | Is it clear in the study what is the “cause” and what is the “effect” (i.e. there is no confusion about which variable comes first)? | Yes | Yes | Yes | 1. Were the two groups similar and recruited from the same population? | Yes | Yes | Yes |
| 2. Were the study subjects and the setting described in detail? | Yes | Was there a control group? | Yes | NO | Yes | 2. Were the exposures measured similarly to assign people to both exposed and unexposed groups? | Yes | Yes | Yes |
| 3. Was the exposure measured in a valid and reliable way? | Yes | Were participants included in any comparisons similar? | Yes | Yes | Yes | 3. Was the exposure measured in a valid and reliable way? | Yes | Yes | Yes |
| 4. Were objective, standard criteria used for measurement of the condition? | Yes | Were the participants included in any comparisons receiving similar treatment/care, other than the exposure or intervention of interest? | Yes | Yes | Yes | 4. Were confounding factors identified? | Yes | Yes | Yes |
| 5. Were confounding factors identified? | Yes | Were there multiple measurements of the outcome, both pre and post the intervention/exposure? | Yes | Yes | Yes | 5. Were strategies to deal with confounding factors stated? | Yes | Yes | Yes |
| 6. Were strategies to deal with confounding factors stated? | Yes | Were the outcomes of participants included in any comparisons measured in the same way? | Yes | Yes | Yes | 6. Were the groups/participants free of the outcome at the start of the study (or at the moment of exposure)? | Yes | Yes | Yes |
| 7. Were the outcomes measured in a valid and reliable way? | Yes | Were outcomes measured in a reliable way? | Yes | Yes | Yes | 7. Were the outcomes measured in a valid and reliable way? | Yes | Yes | Yes |
| 8. Was appropriate statistical analysis used? | Yes | Was follow-up complete and if not, were differences between groups in terms of their follow-up adequately described and analyzed? | Yes | Yes | Yes | 8. Was the follow up time reported and sufficient to be long enough for outcomes to occur? | Yes | Yes | Yes |
|  | | Was appropriate statistical analysis used? | Yes | Yes | Yes | 9. Was follow up complete, and if not, were the reasons to loss to follow up described and explored? | Yes | Yes | Yes |
|  |  |  | | | | 10. Were strategies to address incomplete follow up utilized? | Yes | Not applicable | Not applicable |
|  |  |  |  |  |  | 11. Was appropriate statistical analysis used? | Yes | Yes | Yes |
